# Seeing Ourselves in Others: Understanding and Addressing Biases in Medical School Admissions Processes

**DOI:** 10.5334/pme.1643

**Published:** 2025-01-20

**Authors:** Khadija Ahmed, Tisha Joy, Javeed Sukhera

**Affiliations:** 1 Undergraduate Medical Education, Schulich School of Medicine and Dentistry, Western University, London, Ontario, Canada; 2 Department of Medicine, Schulich School of Medicine and Dentistry, Western University, London, Ontario, Canada; 3 Hartford Hospital and the Institute of Living, Hartford, Connecticut, USA

## Abstract

**Purpose:**

Medical school admissions is a vital area for advancing diversity, equity, and inclusion (DEI). Integrating bias recognition and management (BRM) within the context of admissions is critical in advancing DEI. However, there is a dearth of empirically informed literature on BRM in the admissions context. Therefore, this study sought to explore how individuals involved in admissions decisions process and integrate bias related feedback.

**Methods:**

The authors conducted a qualitative exploratory study using constructivist grounded theory. 21 semi-structured interviews were conducted with various participants in the admissions process at a North American medical school who had participated in bias related training. Participants included medical school faculty, senior medical students, and community volunteers.

**Results:**

Overall, participants expressed diverse perspectives on their personal biases and how these biases impact admissions decisions. Their reflections were shaped by their identities, values, and priorities, which varied based on whether they were faculty members, students, or community members. Participants also highlighted that their biases influenced their perceptions of the ideal admissions candidate, thus influencing their decision-making process. They emphasized the need for more opportunities to engage in dialogue with peers to openly share and discuss how to recognize and manage their biases.

**Conclusion:**

Our study suggests that fostering critical reflection about identity tensions, building and sustaining a community of practice, and facilitating sustained dialogue may provide admissions committees with an evidence-informed, meaningful, and sustained approach to advancing DEI through bias recognition and management.

## Introduction

Advancing diversity, equity, and inclusion (DEI) is a mainstay for medical school admissions. Recruitment of a diverse student body and increasing representation in the physician workforce has benefits for patients and the system at large [1,2]. For example, a more representative workforce improves patient-physician communication and trust [3,4]. In addition, inclusive training environments can improve active thinking, empathetic collaboration, and innovative problem solving [3,4]. Minoritized physicians are also more likely to provide care to underserved communities [5]. Therefore, diversifying the medical workforce is a critical component of addressing health disparities and has been advocated by leading organizations in the United States and Canada, particularly in the context of historical legal decisions that challenge medical schools’ existing approaches to admissions and DEI [6,7,8]. Yet, despite increasing attention to advancing equity and representation, the demographic composition of North American medical students is not commensurate with the aspirations of a more diverse workforce as Black and Indigenous students remain grossly under-represented [9,10].

Although there are several mechanisms to advance DEI through admissions, bias recognition and management (BRM) training has become a mainstay in many settings and has gradually proliferated. BRM directly addresses the role and influence of decision-makers by educating them about how implicit and explicit biases may influence selection processes and admissions decisions. BRM involves a focused and strategic approach to both increasing conscious awareness of biases and fostering behavioral changes [11,12,13,14,15,16]. Current approaches to BRM often include the use of reflection prompts such as the Implicit Association Test (IAT) – a computer-based exercise that measures the strength of associations between concepts or groups (Black and White) and evaluations or stereotypes (good or bad) [17]. Research suggests that educating admissions decision makers about their biases can lead to improved representation of minoritized learners [11]. A study by Capers et al (2017), conducted at the Ohio State University, found that 67% of admissions committee members felt that implicit bias awareness tools may be helpful in reducing bias, 48% were conscious of their individual biases when interviewing candidates in the following cycle, and 21% reported that awareness of their implicit biases impacted admissions decisions the following cycle when the most diverse class in its history matriculated at the Ohio State University College of Medicine [11]. Similarly, a study conducted at the University of Maryland School of Medicine demonstrated that strategies such as implicit bias training for medical school admissions teams resulted in an increased number of underrepresented minorities among medical school matriculants [18]. However, despite the potential for BRM to advance DEI, there are many limitations and challenges with current approaches to BRM in admissions.

Research on BRM in medicine highlights that mitigating biases is often challenging and lacks sufficient evaluation, empiricism and rigor. Bias training often focuses exclusively on increasing knowledge and awareness, without sufficient attention to skill building, context, or sustaining behavioral change [13,16]. One barrier to effective BRM relates to the resistance, defensiveness, and emotions surrounding this topic [19,20,21]. In addition, approaches to BRM within the context of medical school admissions are varied and published literature is limited in scope to group-based training and single time point interventions [11,13]. There has also been a critique of foregrounding implicit bias when more direct naming and transformation of racism within medicine needs to be prioritized [22]. Superficial or performative approaches to bias training may not achieve the intended results and have the potential to adversely influence admissions decisions. Therefore, a more empirical and evidence-based approach to enhancing BRM is needed specific to an admissions context.

Given the challenges and lack of success with current approaches, more research is needed to advance an empirical approach to BRM in an admissions context. Therefore, our study sought to explore how individuals involved in admissions decisions process and integrate feedback about their biases. We hoped to gain a deeper understanding of how current efforts to mitigate biases may achieve intended outcomes, while generating insights on ways to improve diversity and representation through admissions.

## Methods

### Approach and Conceptual Framework

The shortcomings of existing approaches are particularly salient in a medical school admissions context. Lack of attention to contextual influences may create circumstances where learners are left with increased awareness of their potential role in perpetuating bias, without a practical understanding of how they can address their potential biases in their specific context [23,24]. Different individuals involved in varying processes, such as file reviewing or interviewing, may participate in the process with numerous motivations and biases about how they conceptualize the ideal candidate for admission [25]. Therefore, individuals who are involved in the admissions processes can provide insight on their unique context and the role that their personal and professional identity may play in such a context.

Building upon existing research regarding how health professionals process and integrate feedback about their biases, we conceptualized BRM in accordance with Sukhera et al’s integrated theory on BRM [13]. This theory proposes that BRM is part of a transformative learning process that starts with dissonance through feedback seeking, followed by contextually specific role reflection, goal setting, and explicit behavioral change in the context of role modeling and psychological safety within interprofessional teams [13]. Part of the research that contributed to this work involved the use of the Implicit Association Test ( IAT) as an elicitation prompt for interviews that were analyzed through constructivist grounded theory (CGT) [26]. This approach explores social phenomenon through the development of novel theories derived from the knowledge and understanding of researchers and research participants and based on constant comparative analysis [26]. CGT was particularly aligned with our research goal where we sought to learn from the perspectives and experiences of admissions participants.

### Participants and Setting

Consistent with theoretical sampling in CGT [26], we selected research participants for their ability to provide rich narrative data about their experiences as participants within the medical school admissions process. All research participants (n = 21) were recruited from a North American medical school. The participants included medical school faculty, community members, and senior students, who participated in the most recent admissions cycle on a voluntary basis in one of several roles: interviewer, file reviewer, or member of the admissions committee who may be involved in interviews, and file review, and other aspects of admissions decisions and adjudications. To develop an understanding of how these unique roles and contexts could influence feedback about implicit biases, we sought out participants from all aspects of the admissions process. Out of those invited, 21 agreed to participate. 11 participants identified as medical school faculty and of these participants, seven were file reviewers and four were interviewers. Seven participants were community volunteers that included two file reviewers, three interviewers, and two members of the admissions committee. Three participants were senior medical students, which included two interviewers and one member of the admissions committee. Further information about participants and their roles is presented in Table 1. All interviews were conducted between June 2018 and August 2018.

**Table 1:** Participant Title and Role in Medical School Admissions Team at REDACTED.


PARTICIPANT IDENTIFIER	GENDER	TITLE (FACULTY, COMMUNITY MEMBER, STUDENT)	ADMISSIONS ROLE (FILE REVIEWER, INTERVIEWER, COMMITTEE MEMBER)

1	F	Community Member	Committee Member

2	M	Faculty	Interviewer

3	M	Faculty	File Reviewer

4	F	Faculty	File Reviewer

5	F	Student	Committee Member

6	F	Community Member	File Reviewer

7	F	Community Member	Interviewer

8	M	Student	Interviewer

9	F	Community Member	File Reviewer

10	M	Community Member	Interviewer

11	F	Student	Interviewer

12	M	Community Member	Interviewer

13	F	Faculty	File Reviewer

14	M	Faculty	Interviewer

15	F	Faculty	File Reviewer

16	F	Faculty	Interviewer

17	M	Faculty	File Reviewer

18	M	Faculty	File Reviewer

19	F	Faculty	File Reviewer

20	M	Faculty	Interviewer

21	M	Community Member	Committee Member


### Data Collection

One researcher conducted all semi-structured individual interviews virtually via audio/video teleconference, with each interview ranging from 30–60 minutes. Interviews began by reviewing the letter of information and consent and were followed by a demonstration of the online Implicit Association Test (IAT), which was used as a reflective exercise and elicitation prompt similar to previous research [27]. Elicitation techniques serve as stimuli to help researchers obtain the desired insights from participants [27]. In-depth individual interviews allow healthcare researchers to co-construct meaning by revisiting participants’ perceptions of events and experiences [28,29]. Although interviews are commonly employed for elicitation, we hypothesized that relying solely on interviews to uncover implicit biases could be challenging. Therefore, in addition to conducting in-person, semi-structured interviews, we integrated the IAT to enhance the depth of participants’ reflections.

Following completion of the IAT, a discussion guide was used to facilitate conversation with three key open-ended questions: 1) What are your reactions to the online demonstration you just completed? 2) What are some ways that you believe bias may influence you in your role with admissions? 3) How can we help those working in admissions mitigate biases in the admissions process? Discussion encouraged participants to reflect on their own implicit biases, how they perceived bias influenced their admissions role, and their impression of efforts to foster BRM during the admissions cycle. Consistent with CGT, the discussion guide was iteratively revised as the study proceeded and included further questions exploring perceptions of implicit bias training provided to participants during the admissions cycles, challenges faced with recognizing and mitigating implicit biases during the admissions process, and motivation for participating in the admissions cycle. Audio recordings were de-identified and transcribed verbatim before proceeding with analysis. Data collected and participant recruitment ceased when theoretical sufficiency was reached with predictable redundancy emerging in established themes [30].

### Data Analysis and Reflexivity

To support constant comparative analysis, data collection and analysis was conducted simultaneously and iteratively by a team member who independently analyzed the first five transcripts using line-by-line, consolidated coding, and axial coding consistent with CGT. All members of the research team reviewed these selected manuscripts and then participated in coding for the remaining transcripts using NVIVO version 12 (QSR International, London, United Kingdom). Throughout the coding process, the team met at regular intervals to discuss the analysis, identify relationships among codes to develop themes, and theorize about how participants’ experiences inform current understanding about the role of implicit biases in the medical school admissions process. We approached reflexivity consistent with a constructivist orientation including personal, interpersonal, methodological, and contextual domains [31,32]. The research team was comprised of a medical trainee with experience conducting qualitative research (K.A), a clinician and former Associate Dean of admissions with experience spearheading DEI initiatives (T.J), and a psychiatrist and PhD scientist in health professions education with extensive experience in implicit bias research and qualitative research methodology (J.S).

## Results

Overall, participants demonstrated a range of responses regarding their own biases and reflected about how biases influence admissions decisions. Participants contextualized their identities. Values, priorities, and biases were often unique depending on whether the participant was a faculty member, student, or from the community at large. Participant perspectives on how bias influenced their definition of an ideal candidate played a significant role in their decisions. As they reflected on the ongoing process of BRM, participants called for more opportunity to share and dialogue about their biases with peers.

### Role Reflection, Values, and Perceptions of the Ideal Candidate

Participant biases were influenced by their perception of their role within the admissions process, with several noting that their prior experiences and identities influenced how they viewed the process of selecting future physicians. For example, physician participants or medical student participants identified and stratified candidates based on their biases about an ideal colleague or an ideal classmate; however, community volunteers identified the importance of a candidate based on their bias about this individual’s potential as a future physician. One community participant noted:

“I think to recognize the important things… required to be a physician. And I think that’s why…the panel of a med student, a physician, and a community member… is excellent. Because, in my mind I always think of it as the student is looking at the candidate from the perspective of would this person be a good classmate, and the physician is looking at the candidate as could I work with this person as a fellow physician, and then as a community member I’m looking at them as how would I feel if this person were my doctor.” (P7).

Similarly, participants varied in their motivations to participate in the admissions process. Several participants suggested that varied motivations will require individualized strategies for bias mitigation. For example, one participant stated that “understanding” motivations “may be helpful in finding ways to mitigate bias.” This participant also highlighted that conversations about an individual’s motivation could facilitate deeper conversations about bias mitigation (P13). Another stated that bias mitigation:

“… depends on the motivation of the people doing it…I’m assuming most people are doing it because they want the process to be fair and equitable and…they’re doing their best to not be biased. So, in that case, the conversation shouldn’t be difficult…I mean it’s volunteer, so people are doing it because they think it’s important. I assume they’re doing because they think it’s something they can contribute” (P15).

Perceptions of admissions related biases and how to mitigate them also appeared to be influenced by the values espoused by decision makers. Participants noted that biases influence how they define a “good physician” or an “ideal candidate” for admission. The factors that comprise an ideal candidate were unique to participants past experiences and their unique role in the process. For example, a participant who was a file reviewer and identified as a teacher shared that the qualities of leadership they valued came from their professional identity as a teacher, yet these espoused values were not part of the form that was provided to file reviewers to complete their review (P4). Another participant from a medical background stated, “I have come to associate certain characteristics with success in the healthcare field…I have to rely on my bank of experiences” (P18).

Attributes or strengths of preferred candidates also varied in relation to the participants role in the admissions process. For example, file reviewers looked for candidate’s strengths based on a candidate’s ability to articulate their strengths in written form. One stated, “did they articulate their point and reference it back to how they could be a professional?” (P6), while another stated they were looking for “the novel nature of what they say…as opposed to a cookie cutter comment” (P1). Meanwhile, interviewers looked for candidates who could demonstrate that they are “a good listener” through verbal communication and interaction with interviewers (P20).

### Affinity Bias

Most participants felt that their biases were shaped by an in-group preference, or a draw toward applicants who shared similar experiences to themselves. Participants described a natural tendency to “recognize the story is something like yours” and as a result “be more likely to be positively inclined to that candidate” (P15). Such examples of affinity bias and in-group preference varied among participants, with some clarifying that the degree of their affinity bias related to how they perceived their own identity. For example, one participant emphasized that they prioritized applicants who they perceived as under-represented in medicine while reflecting that they could be somewhat biased against applicants who were of a similar identity as them, noting that an applicant with a similar identity, “has to work a little harder for me to think that they need to come in.” (P17). When asked how this type of bias influences admissions decisions, another participant described the role of the alignment between the lived experiences of the applicant and the participant:

“If my experiences are extremely different than the person sitting across the table from me, it might impact the way that I evaluate them. If I thought they weren’t hard working, or that the experiences were just given to them – then, I guess that would mean [they are] not going out of your way to seek learning opportunities, sort of just following the generic algorithm. Then I would tend to be more biased.” (P8).

Some participants also expressed concern with how affinity bias perpetuated potential discrimination within the medical school admissions system, describing that

“…people are seeing themselves in other people like them and there’s already this systemic oppression really, that like is rooted in the system, so of course like these people – and this is an individual level – but they’re kind of choosing people that are similar to them and keeping that idea of systemic oppression really where, you know, and then this results really in having a class of mostly white people” (P11).

Another stated, “if you look historically to medical schools…there’s definitely the idea that ‘like selects like’” (P17).

Participants also suggested that affinity bias was important to consider when mitigating biases in an admissions context. One stated that many admissions decision makers “internalize” and “favour” applicants who are “similar” and that therefore admissions decisions should be made by diverse groups (P6). Another stated:

“It is important to have someone that might be part of your in-group or something like that to be interviewing … I feel like it helps mitigate some of the implicit bias effects…. So, for example when I was interviewing, I’m part of the LGBTQ community and at least two of the people I interviewed outwardly talked about being part of the community. And I think that positively, that was a positive bias for them. Just because, I know it’s a minority in healthcare I know there’s a history of LGBTQ plus people not receiving good health care because of their sexual orientation or their gender identity. So, I think, having more people that can kind of relate to that group can help patients in general…. it’s kind of a different thing in that way I don’t want to admit that I’m being biased because I don’t want to be, but also at the same time it’s hard not to be when you are part of that in-…”(P11).

### Shortcomings of Existing Approaches to Bias Training

Overall, participants appreciated the bias training that was provided to them while expressing skepticism regarding the value that current approaches to training provide. One participant stated, “the way I recall it anyway I don’t recall there being too much extra. I think most people by now have at some point encountered similar bias or bias training or sensitivity training” (P2). Participants suggested that existing approaches to training should be complimented by opportunities for individualized reflection. One stated that individual reflection was “much more impactful than the presentation we had” (P2), and another suggested that didactic training was “insufficient to allow groups to have conversations about what our perceived biases might be and strategies to mitigate that” (P13).

While recognizing the limits of current approaches, participants suggested that building a community of practice to foster sustained dialogue and more reflective conversations was desirable. For example, one participant stated that interaction with others helps decision makers be “more present cognitively” and “emotionally” (P4) and another stated:

“I really believe that in order to open up this entire can of worms and try and mitigate bias as much as possible, we need to have opportunities to talk about it and discuss strategies as a group rather than leaving it to individuals…We may all have the best of intentions, but we’ll have our blind spots…We may be comfortable that we’re taking all the right steps, but we don’t get a lot of feedback” (P13).

Yet, participants also acknowledged the challenge of scheduling and finding dedicated time for group discussions with one sharing, “that’s very difficult to do with…400 interviewers…considering time and schedules” (P7).

## Discussion

In our exploration of how admissions decision makers process and integrate feedback about their biases we learned that participants’ unique identity, values, and role within the process appeared to influence how they perceived their biases and how they could be mitigated. Our findings suggest that empirically informed approaches to advancing BRM in admissions requires critical thinking about identity, affinity, and fostering an individualized approach to reflection. There is a need to shift away from single-session training towards continuous dialogue and engagement, which may be facilitated by fostering a dialogue through a community of practice for those involved in admissions decisions. We believe that our findings can inform an empirically informed and practical framework to foster meaningful and sustained implementation of BRM in admissions.

### Lessons from an admissions context

Our findings highlight shortcomings with existing approaches while drawing attention to contextual nuances in how biases are perceived, how they influence decisions, and how they can be mitigated. Previous research highlights the role of identity in the process of BRM [13,20,33]. Our participants suggested that admissions processes cannot rely on bias education or training alone and must have other processes built into the admissions process to advance DEI. We also found that identity and an individual’s unique role in the admissions process must be attended to. In addition, our participants highlighted that affinity bias must be better understood, confronted, and mitigated through both individual and structural interventions. Therefore, approaches to increase diverse representation in admissions must be complemented by attention to the complexities of affinity bias. Admissions offices should also ensure that stakeholders have sufficient opportunities to listen, learn, reflect, and discuss with others involved in admissions decisions. Ongoing training, coaching, and dialogue can be complemented with individualized reflection and facilitated through a community of practice for diverse groups involved in the admissions process. Such approaches are useful to sustain the impact of singular trainings and foster an iterative cycle of feedback, reflection, and behavior change [13]. Table 2 provides a framework informed by our findings for advancing BRM in admissions.

**Table 2:** Summary of Strategies to Mitigate Bias for Decision Makers in Medical Admissions.


	DESCRIPTION	CURRENT APPROACH	RECOMMENDED APPROACH

CONTEXTUALIZING IDENTITY	Examining power relations and assumptions including individual and societal beliefs and values.	Prompts or triggers such as the IAT are offered to groups to think about how bias influences decisions.	Individuals are invited to critically reflect on their own identities, motivations to be involved in admissions decisions, assumptions about what an ‘ideal’ candidate would look like.

FOSTERING COMMUNITY	Groups of individuals with shared visions, learning together to achieve common goals through regular dynamic interaction	Admissions decision makers are offered training without ongoing coaching, dialogue, or contextually relevant sharing with peers.	Admissions decision-makers regularly interact with, and belong to, a safe and contextually relevant training group where they participate in dialogue about their own biases.

FACILITATING DIALOGUE	Providing spaces for continuous engagement of participants’ whole selves with the goal of enhanced understanding of themselves, each other, and the world	Training sessions include facilitated discussion; however, such discussions are not sustained.	Cultivate an open and supportive training environment where members are encouraged to engage in dialogue to question assumptions and explore new knowledge and perspectives.


### Identity Tensions and the Role of Context and Critical Reflection

Individualized critical reflection is a process that involves examining individual assumptions in the context of broader power relations, including individual and societal beliefs and values [32]. Individualized critical reflection also affords decision makers the opportunity to reflect on the ways in which their unique personal identity may influence their assumptions, inferences, and ultimately their decisions regarding some applicants compared to others. The affinity of one decision maker is likely to vary from others. While the role of affinity bias in selection decisions is well-established [34,35], our findings suggest that not all affinities are applied similarly. For example, decision-makers’ affinity for similarities in socio-economic status influenced their attributions of what would make for an ideal physician differently than their affinity towards an applicant of a similar ethnic background. Therefore, fostering individualized critical reflection shifts away from a “one size fits all” group-based approach, emphasizing components of an individual’s unique identity and the dynamic ways that their identity may influence their potential bias in admissions decisions. Our findings also suggest that fostering individualized critical reflection requires reflection on how an individual’s life experiences that shape biases regarding the ideal of a “good physician.” Existing tools such as reflection prompts for evaluation of inner circle members through various social, cultural, ethnic, and political dimensions, as well as categorization of self-identities with group memberships may be useful [35,36]. Research suggests that there must be regular opportunities for critical reflection through dialogue to allow decision-makers to be more aware of biases during the admissions cycles and thus encourage admissions members to be more mindful of the judgements and decisions they make during the admissions process [35,36]. We recommend incorporating such discussions into meeting structures and framing them as “teaching and learning moments” at the start of committee meetings on a regular basis [37].

### Building a Community of Practice and Fostering Sustained Dialogue

Our findings also suggest that moving from individual critical reflection to sustained skill building and behavior change cannot be achieved on one’s own. Our participants noted that they were diverse and often felt isolated from one another. They shared that being part of a community that shares goals and objectives may facilitate an increased sense of psychological and emotional presence. Interaction with others provides individuals with the opportunity to receive feedback in an empathic manner which minimizes defensive reactions and facilitates further reflection. Any individual involved in admissions is likely not doing such work on a full-time basis, they may participate in a training and then return to their regular working environment. Therefore, an ongoing community can support their ability to reflect, process, and reconcile tensions between how their individual biases relate to the structural and systemic biases around them [33].

Drawing from existing research on BRM, building a Community of Practice (CoP) that is contextually relevant to admissions may have several advantages. Building such communities may enable admission teams to co-construct knowledge and understanding of biases through shared experiences, discussions, and collective responsibility that balance agency and self-efficacy while building trust through relationships [23,36,37,38]. Such relationships allow for effective management of feelings of defensiveness, shame, and guilt – which may be managed by normalizing biases and clarifying the ubiquitous nature of bias in relation to human cognition [13,23]. CoP can also draw attention to structural biases which are reflected in broader institutional policies and practices [39,40]. Therefore, an admissions-specific CoP allows contextually relevant discussions about the role of an admissions committee in relation to DEI values and objectives.

Cultivating a safe CoP within the context of health professions admissions requires collaboration, commitment, and engagement from members of the group. Based on the CoP framework proposed by Snyder and Wenger [41], an essential element of a CoP is a domain that establishes the boundaries and creates a sense of common ground and identity within the community [41,42]. For admissions, strategies for cultivating a CoP require an understanding about what motivates admissions stakeholders to volunteer in admissions processes, as well as an understanding of the shared values upheld by the community. To this end, conducting surveys to understand admissions stakeholders’ values and motivations for participating in the admissions process would be helpful for cultivating a CoP. Consideration must be given to the fact that a large majority of admissions stakeholders participate on a voluntary basis; therefore, developing strategies to encourage involvement and facilitate retention are necessary to cultivate a CoP. Strategies to incentivize involvement and retention may, for example, include professional development credits or compensation for time. Adopting frameworks for successful creation of CoP from fields of health professions education [43] and business [44] may also be helpful.

Closely intertwined with cultivating a CoP, is the concept of continuous dialogue and learning. In contrast to discussion, dialogue refers to engagement of the participants’ whole selves with the goal of enhanced understanding of themselves, each other, and the world [45,46,47]. Dialogue also aims to emphasize interpersonal relationships and trust while enabling confidence to question assumptions and challenge norms [45,46,47]. Dialogue is distinct from group discussion because it tends to be more open ended and generative [45,46,47]. The goal of dialogue is to create opportunities to acknowledge and question assumptions while generating new ways of seeing [45,46,47]. Tools such as the “Time-In” approach have been useful in interrupting bias in admissions teams, by allowing participants to pause conversations, name biased behavior, and engage in dialogue to “call in” expressions of bias [48]. Additionally, given that dialogue is based on interpersonal relationships, cultivating conditions that promote shared values and facilitate positive interactions is critical for a CoP and dialogic forms of learning [45,46,47].

### Limitations

Our sample is limited to participants from one university in Ontario, Canada [49]. Given the differences between university communities across Canada and globally, this study simply provides a framework through which to center implicit bias training. Consideration should be taken to contextualize the findings to make them appropriate for each institution’s local context and needs. In addition, this study was conducted in 2018 and 2019 which was prior to the global COVID-19 pandemic as well as the proliferation of North American initiatives in equity, diversity, and inclusion. Therefore, awareness of the socio-historical context in which the study took place is important when interpreting and considering the findings into the future.

## Conclusion

Improving our knowledge and training around implicit bias in the context of medical school admissions is a key component of enhancing equity. Our study demonstrates that conversations, teaching, and reflection about implicit biases require critical thinking about identity and these discussions may play a role in improving long-term retention and make bias training more cognitively present during admissions cycles.
